# Primary extra nodal Non-Hodgkin's lymphoma of urinary bladder presenting as a bladder tumor: A case report

**DOI:** 10.1016/j.amsu.2020.05.045

**Published:** 2020-06-17

**Authors:** Dr. Namita Bhutani, Dr. Vartika Goel, Dr. Pradeep Kajal, Dr. Devendra Pawar, Dr. Pooja Sharma, Dr. Rajeev Sen

**Affiliations:** a Deptt.of Pathology, PGIMS Rohtak, Haryana, India; b Deptt.of Pediatric Surgery, PGIMS Rohtak, Haryana, India; c Deptt.of Urology, PGIMS Rohtak, Haryana, India

**Keywords:** Cystoscopy, Diffuse large B-Cell lymphoma, Extra nodal, Non-Hodgkin, Urinary bladder

## Abstract

**Introduction:**

Primary non-Hodgkin's lymphoma (NHL) of urinary bladder is an exceedingly rare entity accounting for 0.2% of the primary neoplastic lesions. This tumor has female predominance; with most of the cases seen in middle-aged females. It primarily affects urinary bladder without involvement of the surrounding tissues and lymph nodes. The common presentations include hematuria, dysuria, urinary frequency, nocturia, and abdominal or back pain. The clinical, radiological and endoscopic signs are not very specific. It is diagnosed by its characteristic morphology and immunohistochemical characteristics. The various therapeutic options available are chemotherapy; radiotherapy and surgery used either alone or in combination. Presentation of Case: We hereby report a case of 40 years old man who presented with hematuria as the presenting symptom. On radiology, diffuse thickening of bladder wall was noted, which was biopsied. On histopathology, it was NHL, Diffuse large B cell type. He was treated with chemotherapy (6 cycles of CHOP) and radiotherapy for primary NHL of the bladder and now he is in complete remission.

**Discussion:**

Primary lymphoma of the urinary bladder is exceedingly rare. Non-specific presentation and rarity of this disease pose a diagnostic challenge for both the clinician and the histopathologist. Diagnosis is based upon the characteristic morphology and is supported by immunohistochemical analysis.

**Conclusion:**

All patients with extra-nodal lymphoma need thorough diagnostic work up like nodal lymphomas to arrive at exact staging of the disease to outline subsequent management.

## Highlights

• Primary lymphomas of the urinary bladder are extremely rare.
• Non-specific presentation and rarity of this disease pose a diagnostic challenge for both the clinician and the histopathologist.
• Usually, the diagnosis is one of exclusion. It is made on biopsy with immunohistochemical study and after a negative study of disease extension.

## 1 Introduction

Primary extra nodal non-Hodgkin's lymphomas (NHL) arise from the tissues other than lymph nodes and sites that normally do not contain lymphoid tissues. These comprise about 10–20% of all NHL cases. This entity is heterogeneous and complex in their behavior [1]. The most common sites involved are gastrointestinal tract, head and neck, skin, central nervous system, bone, testis, breast and thyroid. Primary lymphomas of the urinary bladder are extremely rare, representing 0.2% of all extranodal lymphomas and <1% of all bladder tumors [2]. Eve and Chaffey discovered the disease in 1885 [3]. It primarily involves urinary bladder without involving the surrounding tissues, lymph nodes and bone marrow. Females are affected more frequently than males and most of the cases occur in middle-age females. The most common presentations are gross hematuria followed by concomitant urinary tract infection, dysuria, increased urinary frequency and abdominal or back pain. Complications like hydronephrosis, fistulas or involvement of the entire bladder are very rare. The radiological investigations of the urinary bladder reveal submucosal masses: 70% of cases are solitary masses; 20% of cases are multiple masses; and 10% of cases show diffuse bladder wall thickening [1].

The lymphoma of the urinary bladder can be classified into three different categories: (i) primary lymphoma localized to the bladder; (ii) lymphoma presenting in the bladder as the first sign of disseminated disease; (iii) recurrent urinary bladder involvement by lymphoma in patients with a history of malignant lymphoma (secondary lymphoma). The majority of primary bladder lymphomas are low-grade tumors with a good prognosis; the most common type being extranodal marginal zone/mucosa-associated lymphoid tissue (MALT) lymphoma and this typically affects adults who are more than 60 years old. The history of chronic cystitis is commonly associated with this type of tumor. High-grade tumors are rarer, with the most common type being diffuse large B-cell lymphoma (DLBCL) [4]. Usually, the diagnosis of primary lymphoma of the bladder is one of exclusion. It is made on biopsy with immunohistochemical study and after a negative study of disease extension, which includes bone marrow biopsy, computed tomography (CT) and positron emission tomography (PET) to assess for other nodal or extranodal involvement when a possible primary lymphoma of the bladder is suspected. The differential diagnoses of lymphoma of the urinary bladder include urothelial carcinoma with prominent lymphoid infiltrate and undifferentiated carcinoma [3]. The prognostic factors of lymphoma of the urinary bladder include histological subtype and the stage of the tumor. Radiotherapy, chemotherapy and surgical excision are various treatment modalities [1]. Due to its rarity very few cases have been reported in the literature. Herein we describe a case of primary NHL of urinary bladder diagnosed and managed at our institute. The SCARE criteria were utilized for this case report [5].

## 2 Case report

**Fig. 1:**
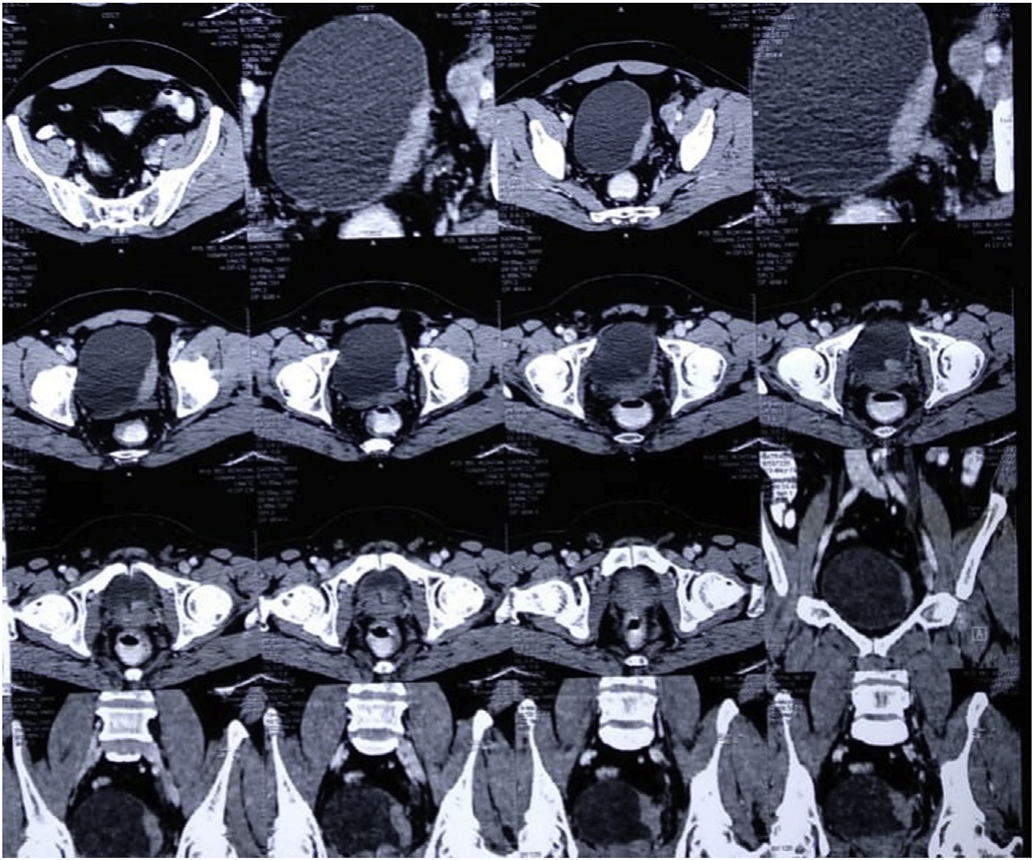
On CT Scan, diffuse thicking of urinary bladder is evident.

A 40-years-old male, farmer by occupation, resident of Rohtak, Haryana (India), presented to urology outpatient department with painless gross hematuria for 2 weeks. There was no history of fever, loin pain, dysuria, urgency or increased frequency of urine. He was a non-smoker. On clinical examination, no pallor or lymphadenopathy was found. Abdominal examination revealed no organomegaly. His routine hematological examination including complete blood counts, coagulation profile, liver function tests, renal function tests and serum electrolytes were within normal limits. Serum biochemical and electrolyte parameters were within normal range. Serology for HIV, HbSAg and HCV were non-reactive. Urine analysis showed hematuria. On ultrasonography and Computed Tomography scan, a diffuse thickening of bladder wall was noted (Fig. 1). The patient was planned for cystoscopy and transurethral resection was performed.

**Fig. 2:**
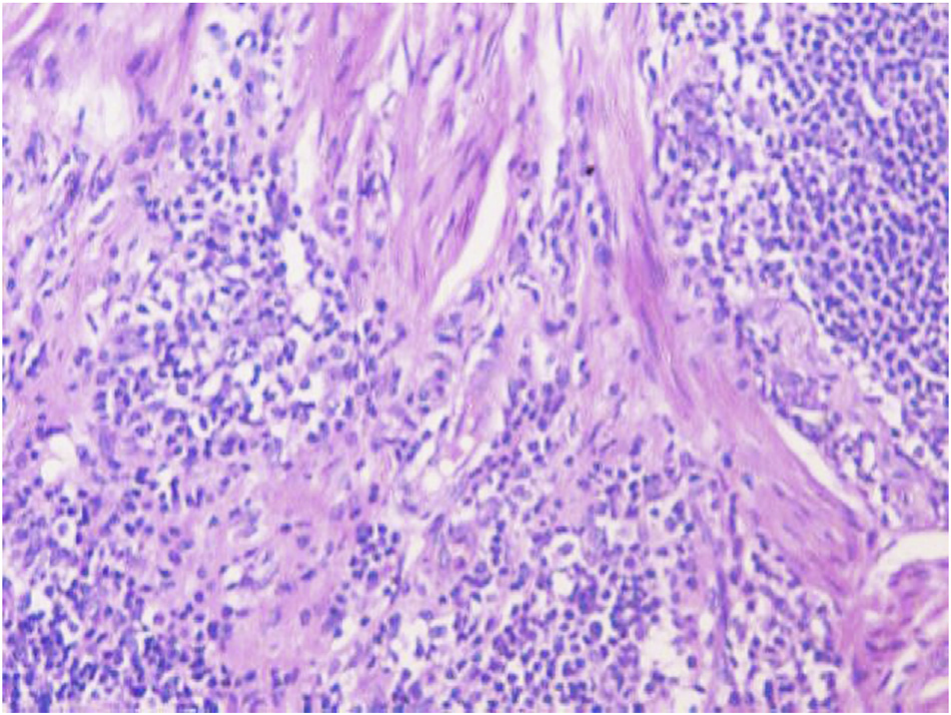
On H&E, tumor cells are seen invading the muscle bundles. (100X).

**Fig. 3:**
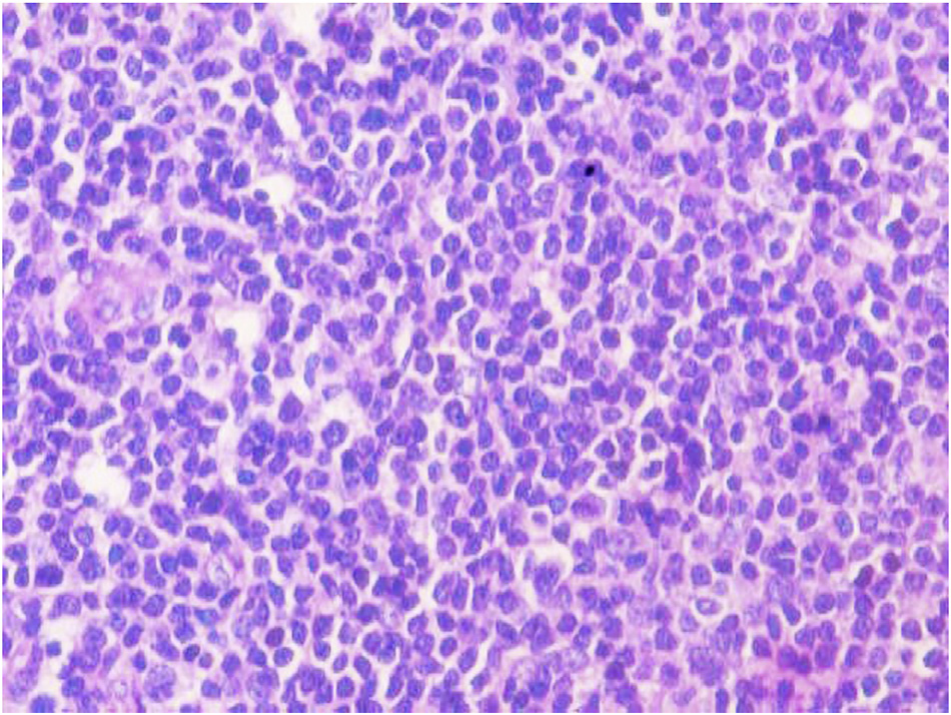
On high magnification, monomorphic population of lymphoid cells (H&E−400X).

**Fig. 4:**
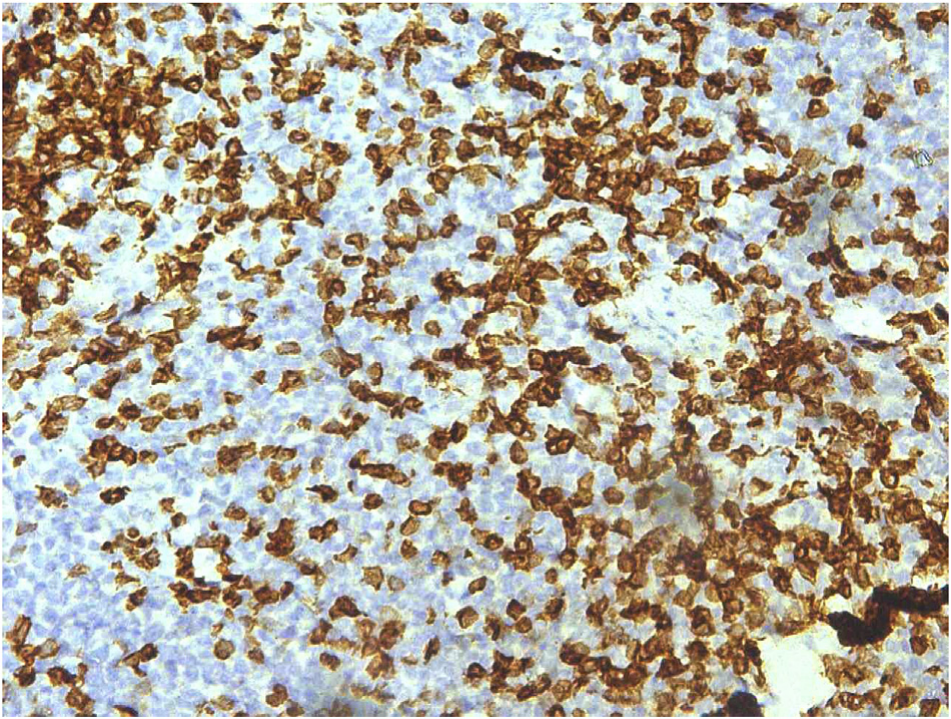
On IHC CD 20 is diffusely and strongly positive (100X).

**Fig. 5:**
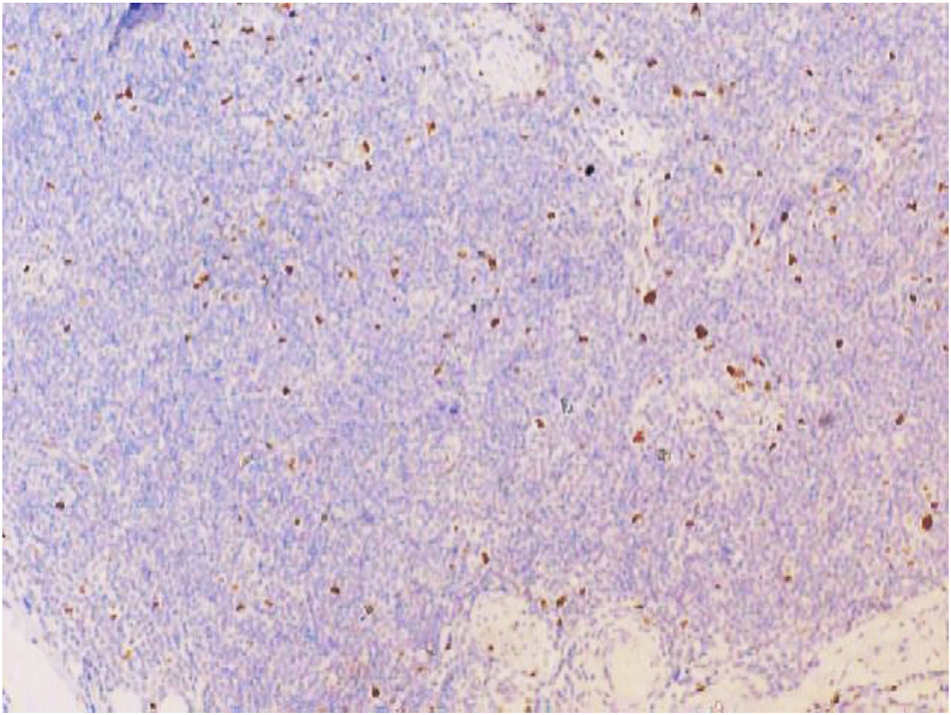
Ki67 labelling index of approximately 30%. (100X).

The biopsy comprised of multiple grey white to grey brown soft tissue pieces measuring together 3x2x1 cm and weighing 4 gm. Histological examination revealed diffuse infiltration of lamina propria by atypical lymphocytes with pleomorphic and hyperchromatic nuclei (Fig. 2, Fig. 3). On immunohistochemistry the tumor cells were strongly positive for CD 20 (Fig. 4) and were negative for Bcl2, CD 5, CD10, CD 23, cyclin D1 and Tdt. The proliferative fraction of cells, as determined by Ki-67 staining was 40% (Fig. 5). Immunohistochemical analyses were negative for cytokeratin and vimentin. Thus, based on histological and immunohistochemical findings, a diagnosis of B cell lymphoma was made.

Due to the highly invasive nature of the tumor, bone marrow (BM) biopsy was performed which showed no evidence of leukemia or lymphoma. Further investigations including chest radiography and CT scans of his chest, abdomen and pelvis were performed which neither showed any lymphadenopathy nor any organomegaly. No metastatic lesions were found. Thus, the final diagnosis was primary B cell lymphoma of the bladder. The disease was classified stage IE according to the Ann Arbor Staging system and the patient had a score of 0 by using the international prognostic index (IPI). Considering age, general condition of the patient and stage of tumor, the patient underwent six cycles of R–CHOP regimen (Rituximab, Cyclophosphamide, Vincristine, Doxorubicin, Prednisolone) followed by radiotherapy of 30 Gy in 20 fractions 1 month after completion of all cycles of chemotherapy. There were few side effects of chemotherapy in patient which included nausea, vomiting, weight loss and fatigue. He showed good tolerance and initial response to this treatment. Thus far, PET CT scans have shown no abnormal uptake, indicative of effective treatment. Now the patient is in complete remission without evidence of local or distant recurrence on routine follow up since 2 years. His urine cytology, cystoscopy as well as urography were performed 3 months postoperatively and after every 3 months interval for a period of 2 years. All these were negative and a repeat CT scan performed after 2 years was negative too. Currently, patient is free from disease and is doing well.

## 3 Discussion

In 1885, Eve and Chaffey first described primary malignant lymphoma of urinary bladder [3]. Usually lymphoid tissue is not observed in urinary bladder, so it is relatively uncommon site for primary extra nodal NHL accounting for less than 1% of neoplasms in urinary bladder and 0.2% of cases with extra nodal lymphoma [2]. The most common sites involved are gastrointestinal tract, head and neck, skin, central nervous system, bone, testis, breast and thyroid [1].

Primary NHL of urinary bladder commonly affects women with female to male ratio of 6.5:1.Various presenting symptoms include intermittent hematuria, dysuria, nocturia, urinary frequency, pain in lower abdomen and frequent or recurrent urinary tract infections [6]. The etiology of primary NHL of the bladder has not been elucidated, because of the rarity of this neoplasm. According to some authors, lymphoma may be secondary to chronic cystitis. Tumor associated with chronic ystitis may originate in the lymphocytes of the submucosa of bladder because of inflammatory response. Other authors have raised the possibility of a residual embryonic cloaca, source lymphoid proliferation in adulthood [7].

Cohen et al. stated that malignant lymphoma of the urinary bladder can be classified into one of three different clinical groups as follows: (i) Primary lymphoma localized to the bladder; (ii) Lymphoma presenting in the bladder as the first sign of disseminated disease (nonlocalized lymphoma); (iii) Recurrent urinary bladder involvement by lymphoma in patients with a history of malignant lymphoma (secondary lymphoma). Primary bladder lymphomas are of two types: Mucosa associated lymphoid tissue (MALT) and Diffuse large B cell type of lymphoma (DLBCL). MALT type lymphoma is more common. Secondary involvement of the bladder by systemic lymphoma has been documented in 10–20% of NHL patients [8].

Bladder lymphoma usually locates at the base and the trigone of the bladder as a sessile mass with normal urothelium. Commonly such lesions are initially diagnosed as urothelial carcinoma of the bladder. CT scans and MRI examination usually do not add much information for diagnosis due to their low sensitivity. But cystoscopy, histological examination and immunohistochemistry play major role for diagnosis of the disease. On cystoscopy, these tumors are visualized as well-defined intravesicular masses typically located at the dome or the lateral walls of the bladder [2]. The DLBCL is characterized by a proliferation of atypical lymphoid cells. The tumor cells are of large size and often resemble normal centroblasts or immunoblasts. These cells express CD20, CD79a and bcl-2. The positivity of CD10, CD5 and Bcl6 remains variable. The differential diagnosis is essentially a poorly differentiated carcinoma; melanoma, burkitt's and Hodgkin's lymphoma; underlining the interest of an immunohistochemical panel in order to confirm the diagnosis. Immunohistochemical staining for B-cell type lymphomas routinely test positive for CD19, CD20 and CD21. Furthermore, low grade lymphomas routinely test positive for CD20, CD21, and CD43 cell markers, while high grade lymphomas are correlated with CD3, CD20, and CD3 [9]. Once the type of bladder lymphoma has been identified, it is imperative to make the distinction between an indolent, low grade neoplasm versus an aggressive, high grade malignancy in order to initiate the most appropriate treatment regimen for that patient. If determined to be a high grade tumor, it is imperative to exclude systemic involvement; this can be done with a BM biopsy and a PET scan [9].

There are different therapeutic strategies available for primary extra-nodal NHL like radical cystectomy, radiotherapy and chemotherapy depending upon the clinical behavior of tumor, risk of disease progression, life expectancy and general condition of the patient. For MALT lymphoma it is recommended that a less systematically toxic therapy like TURBT be attempted first, followed by chemotherapy, radiation, or combination therapy. No recommendations on lesion size have been published for qualifications for TURBT. Therefore following criteria set for other bladder tumors could be used; TURBT has proven effective in urothelial bladder cancer that has yet to invade the musclaris propria (stage Ta, Tis, and T1). As for the type of chemotherapy, all of the regimens (R–CHOP, CHOP, ChlVP, ChlD) were successful in treating low grade bladder lymphomas; R–CHOP was reported by the literature to be used most frequently. DLBCL is an aggressive lymphoma and without treatment, leaves the patient with a prognosis of a matter of months. Chemotherapy is currently the preferential treatment due to its ability to treat early systematic disease which has not been detected. The chemotherapy regimen of R–CHOP (ritoximab, cyclophosphamide, duanorubacin, vincristine, prednisolone) has documented success in treating both low and high grade primary bladder lymphomas as either solitary treatment or combination therapy. Rituximab has been shown to help overcome the chemotherapy resistance seen in patients with BCL-2 over expression by inducing cell apoptosis through the BCL-2 regulated mitochondrial pathway increasing; the survival rate of patients with DLBCL on CHOP chemotherapy by 10–15% with very little additional toxicity.Surgical techniques such as a cystectomy are recommended for any invasive transitional cell carcinoma that have spread into the muscular layer, however there are no clear surgical recommendation for bladder lymphomas and surgical modalities have been disputed in the literature due to the morbidity and mortality associated with these procedures [10,11].Radiotherapy can be used in first intention especially in low grades or in the adjuvant setting after resection but cannot constitute a standard treatment [11,12]. Indeed, its side effects are frequent and managing local recurrence after irradiation is very complicated. The optimum follow-up protocol for patients treated for primary lymphoma of the bladder has yet to be determined. Follow-up evaluations should take place every 3 or 6 months for the first 2 years and yearly thereafter, as is the case for transitional cell carcinoma. It must include at least ultrasound examination and cystoscopy. The prognosis is difficult to be determined given the rarity of primary NHL of the bladder. However, patients with high-grade lymphoma should be considered to have systemic disease and therefore, the IPN can be used to predict prognosis. The factors that should be taken into consideration to outline the management of such patients are age, performance and physiologic status of the patient, stage of tumor and International Prognostic Index (IPN). Calculating IPN that depends on age, stage, serum lactate dehydrogenase, number of extra nodal sites involved and performance status helps to determine the prognosis of the patient.

The indexed case has been highlighted for its rarity. Even if the condition is rare, possibility of malignant lymphoma should be always kept in mind for differential diagnosis of bladder tumor. Because once diagnosed there is chance of excellent complete remission of the lymphoma resulting in good prognosis. As there are no clear therapeutic guidelines for managing such cases, present case will add to the present literature.

## 4 Conclusion

Primary lymphoma of the urinary bladder is exceedingly rare. Non-specific presentation and rarity of this disease pose a diagnostic challenge for both the clinician and the histopathologist. Diagnosis is based upon the characteristic morphology and is supported by immunohistochemical analysis. Radiotherapy and chemotherapy are useful and effective in high grade and locally advanced tumor and it seems to give good results. All patients with extra-nodal lymphoma need thorough diagnostic work up to arrive at exact staging of the disease to outline further management.

## Declaration of competing interestCOI

The manuscript has been read and approved by all the authors, is original and honest work and has not been sent to any other journal for publication and there is no conflict of interests between the authors. Kindly consider it for publication in your esteemed journal.

## Provenance and peer review

Not commissioned, externally peer reviewed.
